# Cost-effectiveness of capivasertib plus abiraterone for PTEN-deficient metastatic hormone-sensitive prostate cancer in the United States

**DOI:** 10.1371/journal.pone.0353373

**Published:** 2026-10-26

**Authors:** Glen J. Weiss

**Affiliations:** Department of Medicine, UMass Chan Medical School, Worcester, Massachusetts, United States of America; Hospital Infantil de Mexico Federico Gomez, MEXICO

## Abstract

**Background:**

On June 12, 2026, the US Food and Drug Administration (FDA) approved capivasertib (Truqap; AstraZeneca) in combination with abiraterone and prednisone for adult patients with metastatic androgen pathway modulation-naive or -sensitive (mAPMN/S) prostate cancer, corresponding clinically to metastatic hormone-sensitive prostate cancer (mHSPC), that is PTEN-deficient as detected by an FDA-authorized test. The pivotal CAPItello-281 trial demonstrated a statistically significant radiographic progression-free survival (rPFS) benefit (hazard ratio [HR] 0.81; p = 0.034) but a nonsignificant and immature overall survival (OS) benefit (HR 0.90; p = 0.401). Capivasertib carries a publicly available list price of approximately $23,762 per 28-day cycle.

**Objective:**

To estimate the incremental cost-effectiveness of capivasertib plus abiraterone and prednisone versus abiraterone and prednisone alone, both with androgen deprivation therapy (ADT), from the perspective of a US third-party payer.

**Methods:**

A three-state partitioned survival model (PSM) was developed with a lifetime horizon (40 years), 4-week cycle length, and 3% annual discount rate. Individual patient-level survival data were reconstructed from published Kaplan-Meier curves via the Guyot algorithm. Given OS immaturity and AIC differences of less than 1 point across six parametric families, a preregistered proportional hazards (PH) base case was applied: the trial OS HR of 0.90 was applied to the placebo parametric fit. Generic abiraterone, ADT, companion diagnostic, and monitoring costs used National Average Drug Acquisition Cost (NADAC) or Medicare reimbursement schedules; capivasertib used a publicly available list-price proxy, and the OS hazard ratio was sampled lognormally in PSA. Uncertainty was characterized via one-way sensitivity analysis, eight pre-specified OS extrapolation scenarios, four scenario analyses, and probabilistic sensitivity analysis (PSA; n = 10,000). Reporting followed CHEERS 2022 standards.

**Results:**

Capivasertib accrued 4.103 discounted QALYs at a total discounted cost of $1,455,507, versus 3.703 QALYs at $207,230 for abiraterone alone. Incremental cost was $1,248,277 and incremental QALYs 0.400, yielding an ICER of $3,120,720 per QALY. The probability of cost-effectiveness was 0% at willingness-to-pay thresholds of $100,000, $150,000, $200,000, and $500,000 per QALY. The most favorable OS extrapolation scenario (log-normal) yielded an ICER of $1,359,445 per QALY. The ratio of mean incremental cost to mean incremental QALY across PSA iterations was $3,148,744 per QALY; because a minority of iterations (13.54%) yielded a negative incremental QALY, the distribution of individual iteration ICERs is uninformative, and uncertainty was characterized using the cost-effectiveness acceptability curve and plane.

**Conclusions:**

At current list pricing, capivasertib plus abiraterone is not cost-effective for PTEN-deficient mHSPC from a US payer perspective. The result is structurally driven by high incremental drug acquisition cost and small incremental QALY gain under immature and nonsignificant OS data. Mature OS data and a major net-price reduction would likely be required for cost-effectiveness.

## Introduction

Prostate cancer is the most commonly diagnosed solid tumor among US men [1]. Patients with metastatic hormone-sensitive prostate cancer (mHSPC), termed metastatic androgen pathway modulation-naive or -sensitive (mAPMN/S) prostate cancer in the current FDA label, face a median overall survival ranging from approximately 44 months in high-volume de novo disease to more than 10 years in low-volume metachronous disease [1]. Abiraterone acetate plus prednisone combined with ADT has become a standard backbone of care for mHSPC following the LATITUDE and STAMPEDE trials [2,3]. Chemohormonal therapy with docetaxel plus ADT also demonstrated a survival advantage over ADT alone in the CHAARTED trial [4], and contemporary practice increasingly favors treatment intensification including ARPI-based doublets and ARPI-plus-docetaxel triplets. Despite these advances, mHSPC remains incurable and subgroups defined by adverse molecular features continue to experience poor outcomes.

Loss of the phosphatase and tensin homolog (PTEN) tumor suppressor gene, present in approximately 25% of patients with mHSPC, activates the PI3K/AKT signaling pathway and confers an independent proliferative drive that androgen receptor pathway inhibitors (ARPIs) may not fully suppress, providing a biologic rationale for AKT-pathway inhibition; PTEN deficiency is associated with inferior outcomes compared with PTEN-intact disease [5].

Capivasertib is a first-in-class oral AKT inhibitor administered at 400 mg twice daily on a 4-days-on/3-days-off schedule [6]. The FDA approved capivasertib in combination with fulvestrant for HR-positive, HER2-negative advanced breast cancer in November 2023 [7]. On June 12, 2026, the FDA granted supplemental approval for capivasertib in combination with abiraterone and prednisone for PTEN-deficient mHSPC as identified by the companion VENTANA PTEN (SP218) RxDx Assay [8]. This approval was based on CAPItello-281, a randomized, double-blind, placebo-controlled phase III trial of 1,012 patients [5]. The trial met its primary rPFS endpoint (HR 0.81, 95% CI 0.66 to 0.98; p = 0.034; median 33.2 versus 25.7 months) but OS was immature and nonsignificant at the time of primary analysis (HR 0.90, 95% CI 0.71 to 1.15; p = 0.401; 26.4% maturity). The FDA Oncologic Drugs Advisory Committee voted 7–1, with 1 abstention, in favor of the risk-benefit profile on April 30, 2026 [9].

Capivasertib’s publicly available list price is approximately $23,762 per 28-day cycle [10]. The combination of a modest rPFS benefit, nonsignificant and immature OS, and high drug acquisition cost creates a challenging cost-effectiveness profile. No published US cost-effectiveness analysis for capivasertib in mHSPC existed at the time of this analysis. This study presents a preregistered PSM estimating the cost-effectiveness of capivasertib plus abiraterone and prednisone versus abiraterone and prednisone alone (both with ADT) from a US third-party payer perspective.

## Materials and methods

### Model overview and structure

A three-state PSM was developed in R (version 4.4) using the flexsurv package [11], following ISPOR-SMDM modeling good research practices [12] and reported per CHEERS 2022 [13]. Health states comprised PFS, post-progression survival (PPS), and dead. State membership at each 4-week cycle was defined as: P_PFS(t) = S_rPFS(t); P_Dead(t) = 1 minus S_OS(t); P_PPS(t) = S_OS(t) minus S_rPFS(t), floored at zero. Where S_rPFS(t) exceeded S_OS(t) at any cycle, S_rPFS(t) was set equal to S_OS(t). A half-cycle correction was applied throughout. Time horizon: 40 years (lifetime). Annual discount rate: 3% for both costs and outcomes, per Second Panel on Cost-Effectiveness recommendations [14]. Perspective: US third-party payer (healthcare costs only; no productivity or indirect costs). The analysis was preregistered on OSF prior to any cost-effectiveness calculations (https://doi.org/10.17605/OSF.IO/68R2J). State-occupancy traces for each arm are shown in Fig 1.

**Fig 1:**
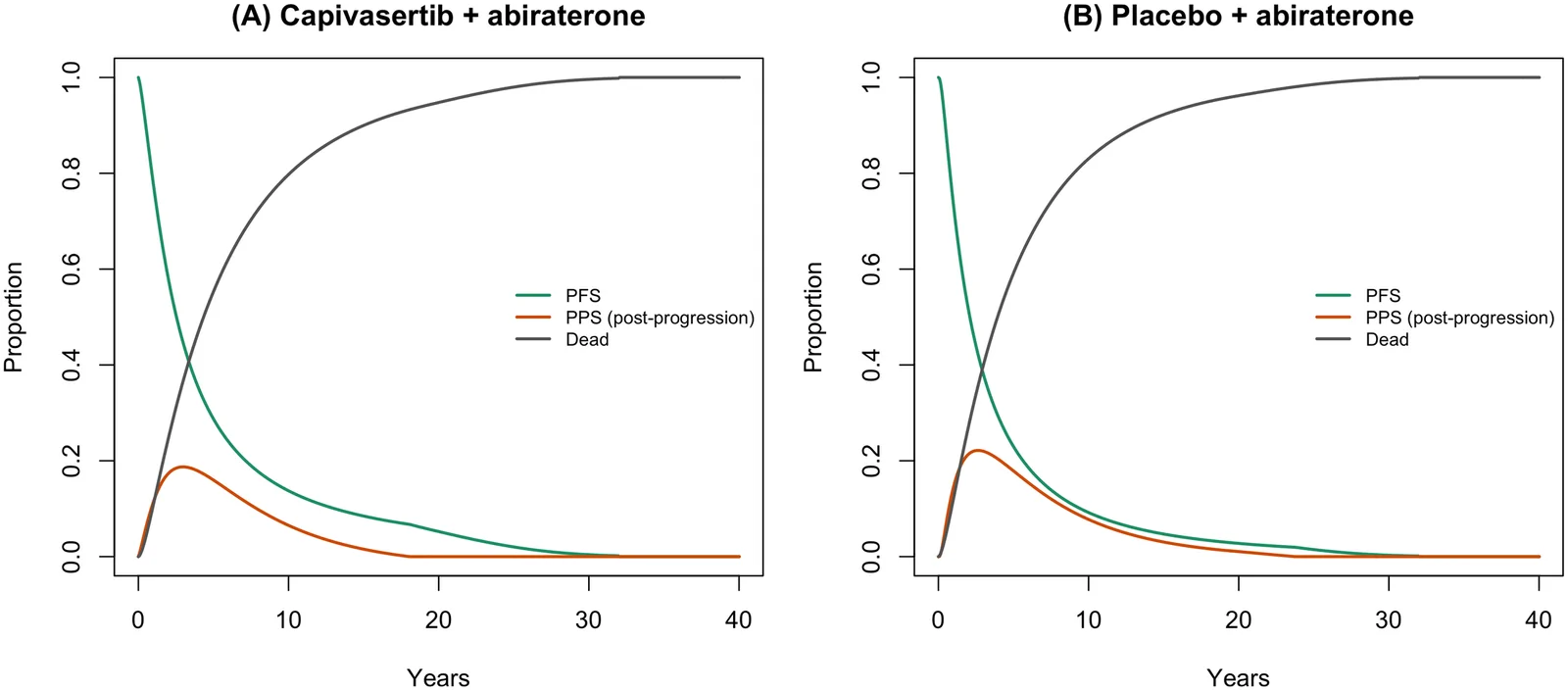
Partitioned survival model state-trace plots for capivasertib arm (Panel A) and placebo arm (Panel B) over a 40-year lifetime horizon. PFS = progression-free survival; PPS = post-progression survival.

### Clinical inputs and survival modeling

Efficacy data were sourced from CAPItello-281 (NCT04493853), published by Fizazi et al. [5]. Individual patient-level survival data were reconstructed fromthe published CAPItello-281 Kaplan-Meier curves for rPFS and OS (Fizazi et al. [5], Figs 2A and 3A) using the Guyot algorithm [15], implemented via the IPDfromKM package in R. Curve coordinates were obtained by automated color-channel (HSV) segmentation of the published figure images using OpenCV, with axis calibration derived from the printed axis ticks, and numbers at risk were transcribed from the published at-risk tables. The full image-to-coordinate-to-pseudo-IPD pipeline is deposited in the OSF project repository (https://doi.org/10.17605/OSF.IO/EWXQT) and reproduces the coordinates used here from the figure images. Reconstruction recovered the published arm sizes exactly (507 and 505). For each curve, the root-mean-square deviation between the reconstructed Kaplan-Meier step function and the digitized input coordinates was 0.01 or less (maximum point deviation 0.10), confirming faithful inversion; reconstructed medians (rPFS 33.3 and 25.7 months) corresponded closely to the published medians (33.2 and 25.7 months), with a maximum deviation of 0.15 months.

Six parametric distributions were fitted by maximum likelihood to each of the four reconstructed datasets (rPFS capivasertib, rPFS placebo, OS capivasertib, OS placebo): exponential, Weibull, log-normal, log-logistic, Gompertz, and generalized gamma. Selection criteria were AIC (primary), BIC (secondary), and biological plausibility, defined as S(40 years) < 0.01. For rPFS, per-arm best-AIC selection was applied; tail plausibility was assessed, but because rPFS curves were subsequently bounded by OS within the partitioned survival model (S_rPFS capped at S_OS each cycle), the best-AIC rPFS capivasertib fit (log-logistic) was retained despite an unconstrained S(40 years) of 0.0243, as its tail is structurally bounded and cannot contribute implausible residual survival. Background mortality was applied as a floor using sex-specific 2022 US male CDC life tables [16], implemented at each cycle as the maximum of the parametric hazard and the age-matched general-population hazard. The modeled cohort entered at the CAPItello-281 median age of 68 years; age advanced by cycle, and the same life-table floor was applied identically to both arms via the OS function. Reconstructed Kaplan-Meier curves with the selected parametric extrapolations overlaid are shown in Fig 2.

**Fig 2:**
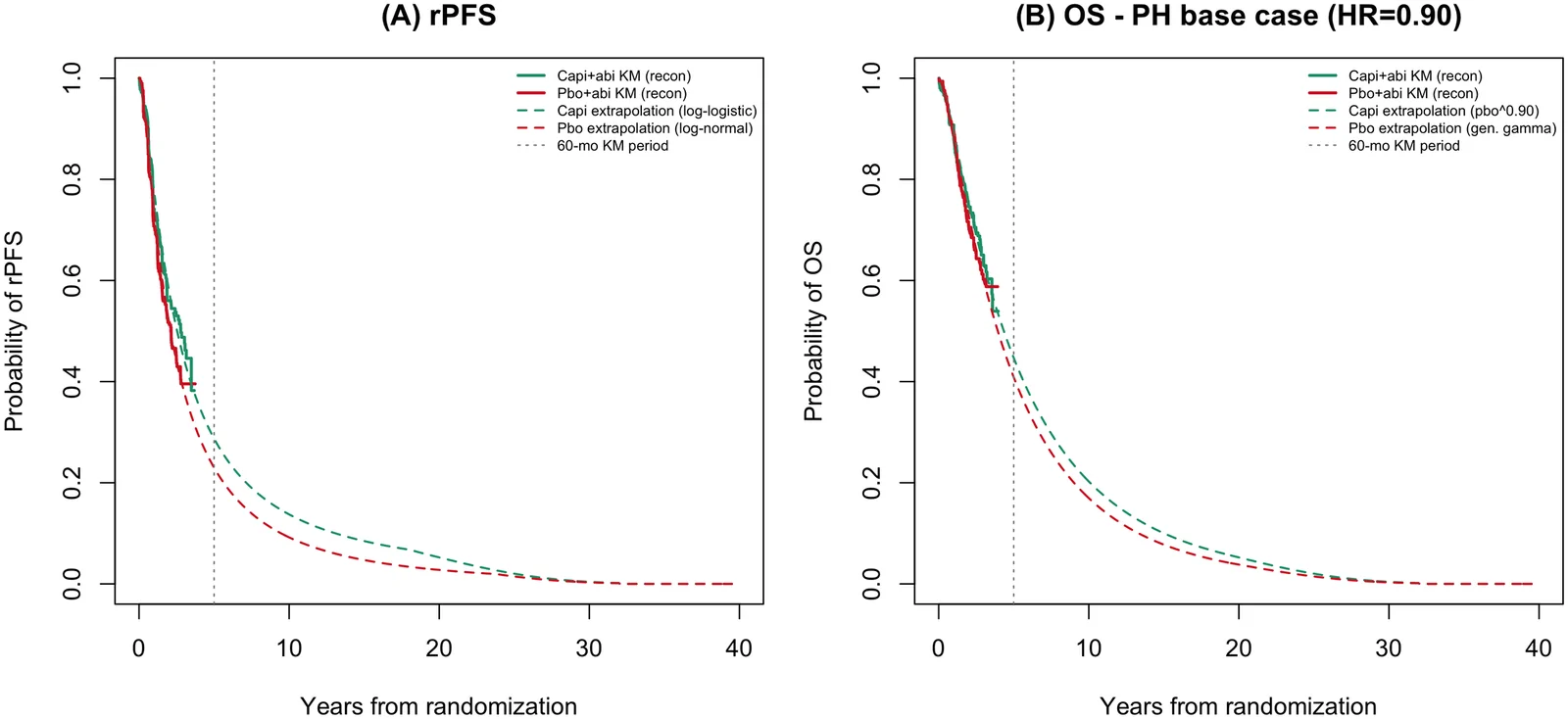
Reconstructed Kaplan-Meier curves with parametric extrapolations for rPFS (Panel A) and OS under the proportional hazards base case (Panel B). Capivasertib, green; placebo, red. Solid lines, reconstructed Kaplan-Meier estimates; dashed lines, selected parametric extrapolations to 40 years; dotted vertical line, end of the 60-month observed period.

For OS, independent per-arm best-AIC fitting produced a clinically implausible inversion driven by a noisy terminal Kaplan-Meier step (n approximately 7 in the capivasertib arm), yielding the capivasertib arm (Gompertz, 40-year restricted mean survival 54.6 months) extrapolating to worse survival than placebo (log-logistic, 40-year restricted mean survival 84.4 months). AIC differences across all OS distributions were less than 1 point (exponential 1404.7, Weibull 1404.4, Gompertz 1404.2), providing no statistical basis for preferring any single family. A proportional hazards base case was pre-specified in the OSF preregistration prior to any ICER calculations: the best-AIC plausible-tail distribution was fitted to the placebo OS arm, and the capivasertib OS curve was derived by applying S_capi(t) = S_placebo(t)^0.90. This approach preserves the trial OS point estimate under a proportional hazards assumption while avoiding the unstable independent-arm tail artifact documented in the preregistration [17,18]. All six single-distribution per-arm OS scenarios plus the per-arm best-AIC artifact scenario are reported for transparency in Table 1.

**Table 1:** OS Extrapolation Scenario Analyses.

OS scenario	Incremental cost	Incremental QALY	ICER ($/QALY)	dOS (mo)
PH base case (HR = 0.90) [BASE]	$1,248,277	0.400	$3,120,720	7.2
Exponential (both arms)	$1,247,697	0.382	$3,263,987	6.7
Weibull (both arms) [common Weibull, independent arms]	$1,251,106	0.763	$1,639,963	16.1
Log-normal (both arms)	$1,389,847	1.022	$1,359,445	24.4
Log-logistic (both arms)	$1,356,609	0.873	$1,553,577	20.0
Gompertz (both arms)	$1,036,942	0.308	$3,367,611	5.3
Gen. gamma (both arms)	$1,047,839	−0.231	-$4,527,893	−8.6
Per-arm best-AIC: Gompertz capi + Log-logistic pbo (artifact – transparency)	$907,764	−0.737	-$1,231,624	−21.5

Negative ICER denotes capivasertib dominated (immature-data distribution artifact, reported for transparency). Data repository: https://doi.org/10.17605/OSF.IO/EWXQT.

Treatment stopping rules: capivasertib and abiraterone were assumed to be discontinued at rPFS progression. Full dose intensity was assumed for both arms throughout PFS. CAPItello-281 reported that 58% of capivasertib-arm patients remained on treatment for 12 months or longer and 35% for more than 2 years, supporting sustained exposure during PFS; however, dose reductions (32%) and interruptions (65%) were common, so the full-dose assumption is conservative against capivasertib and may overestimate its drug acquisition cost relative to real-world exposure. Patients in the PPS state received post-progression costs only; no active capivasertib was modeled post-progression.

### Costs

All costs are expressed in 2025 US dollars. Capivasertib list price was $23,762 per 28-day cycle (64 tablets; 4-days-on/3-days-off schedule), sourced from a publicly available price database [10]. This is a list-price proxy; actual net payer costs may differ due to confidential rebates, which cannot be quantified in the absence of disclosed net price. Generic abiraterone cost was $101 per 28-day cycle (CMS NADAC, April 2026) [19], applied to both arms during PFS. Prednisone 5 mg once daily: $15 per 28-day cycle. Leuprolide acetate 7.5 mg (1-month depot) (ADT backbone, both arms): $176.45 per month (Medicare Part B ASP, HCPCS J9217, Q1 2026) [20], applied to both arms during PFS. The VENTANA PTEN (SP218) RxDx Assay companion diagnostic: one-time cost of $107.55 (CPT 88360, 2026 Medicare Physician Fee Schedule) [21], capivasertib arm only at cycle 1. Post-progression costs for the PPS state (both arms): $9,652 per 28-day cycle ($10,492 per month), derived from the published mean all-cause monthly cost of metastatic castration-resistant prostate cancer of $9,717 per patient-month (2022 US dollars) reported by Kaye et al. [22], inflation-adjusted to 2025 US dollars using the US Bureau of Economic Analysis Personal Consumption Expenditures health care price index (factor 1.0798). Fasting glucose (CPT 82947) and HbA1c (CPT 83036) monitoring was included as a capivasertib-arm cycle-level cost during PFS, reflecting the prescribing information schedule (fasting glucose at weeks 1, 2, 4, 6, and 8 then monthly, and HbA1c every 3 months) [6]; unit costs were 2026 Medicare Clinical Laboratory Fee Schedule allowables (CPT 82947 $3.93; CPT 83036 $9.71; effective 2026-01-01) [21]. Adverse event management costs for grade 3 or higher hyperglycemia, diarrhea, and cutaneous reactions were applied to the capivasertib arm only at incremental rates derived from CAPItello-281 safety data [5] and conservative one-time event-management assumptions, varied in OWSA and PSA; because these costs were small relative to drug acquisition costs, they did not materially affect conclusions.

### Health state utilities

Health state utilities were sourced from the EQ-5D-5L analysis of the TITAN trial (apalutamide in metastatic castration-sensitive prostate cancer) by Agarwal et al. [23], a contemporary mHSPC population with comparable disease characteristics. The PFS state utility (0.80; sensitivity range 0.72 to 0.85) was taken as the on-treatment progression-free value reported in TITAN, and the PPS state utility (0.63; range 0.58 to 0.75) as the post-progression (progressed-disease) value reported in TITAN; both were applied equally to both arms in the base case. Adverse event disutilities for grade 3 or higher hyperglycemia, diarrhea, and cutaneous reactions were applied to the capivasertib arm as one-cycle decrements drawn from the standard oncology toxicity-disutility literature (Lloyd et al. [24]; Nafees et al. [25]). A pre-specified exploratory scenario (S2) applied an additional PFS utility decrement of 0.04 to the capivasertib arm to reflect the numerically faster time to clinically meaningful FACT-P physical well-being decline reported in the CAPItello-281 PRO analysis by George et al. [26]; this decrement was not derived from a formal EQ-5D mapping and is labeled exploratory accordingly.

### Model validation

Internal validity was confirmed by verifying that all state-occupancy probabilities summed to 1.0 at every cycle, that the PPS fraction remained non-negative after applying the rPFS floor, and that survival functions were monotonically non-increasing. Face validity was assessed by comparing reconstructed rPFS medians with published CAPItello-281 values; reconstructed medians (33.3 and 25.7 months) corresponded to the published values (33.2 and 25.7 months) within 0.15 months. Extreme-value tests were performed by setting all cost parameters to zero and confirming that the model produced zero costs with nonzero QALYs, and by setting OS equal to rPFS and confirming zero PPS occupancy. Background mortality was validated by confirming that projected survival curves did not exceed age-matched US general population survival at any cycle.

### Ethics statement

Ethics approval and informed consent were not applicable. This study used only previously published aggregate trial data and publicly available cost data, and no human subjects were involved.

### Use of artificial intelligence tools

Two Anthropic tools were used in the preparation of this work: Claude (Opus-class large language model) and Claude Code. Claude Code assisted the author in writing and debugging the R analysis pipeline that the author specified: reconstruction of individual patient data from published Kaplan-Meier curves (IPDfromKM, flexsurv), the partitioned survival model, the probabilistic and one-way sensitivity analyses, the scenario analyses, and figure generation. Claude assisted with manuscript structuring, language editing, consistency checking against CHEERS 2022 reporting items, and reconciliation of arithmetic between the model outputs, tables, and text. The author defined the model structure, all parameter values and their sources, and every analytical decision; no clinical or economic input value was generated or retrieved from the open web by an AI tool. All numerical results were produced by the analysis code, not by a language model, and the author verified every reported figure against the model engine output and every cost input against its primary source. The author takes full responsibility for the content, accuracy, and integrity of the work. The analysis code is openly available in the OSF repository.

### Sensitivity and scenario analyses

OWSA varied each parameter individually across its pre-specified range while holding all others at base-case values. A tornado diagram was generated for the 12 parameters with greatest influence on net monetary benefit (NMB) at $150,000 per QALY; NMB was used rather than the ICER because incremental QALYs approach and cross zero under some parameter values, rendering ICERs unstable. Eight pre-specified OS extrapolation scenarios were run in the full PSM (Table 1). Four additional scenario analyses were conducted: S1, brand abiraterone list price (e.g., Zytiga or Yonsa) instead of generic NADAC; S2, exploratory PRO-informed PFS utility decrement of −0.04 in the capivasertib arm; S3, 10-year time horizon; S4, WTP threshold of $200,000 per QALY. Uncertainty in the OS hazard ratio itself was characterized through the eight OS extrapolation scenarios and through lognormal sampling of the trial OS HR (point estimate and 95% CI) in PSA, rather than as a separate one-way scenario. PSA used 10,000 Monte Carlo iterations: drug costs sampled from gamma distributions (CV 0.10); utilities from beta distributions (CV 0.10); AE rates from beta distributions derived from CAPItello-281 confidence intervals; AE costs from gamma distributions (CV 0.20); survival model parameters from multivariate normal distributions on log-scale flexsurv coefficients using the full variance-covariance matrix (preserving within-model parameter correlations); and the OS hazard ratio sampled lognormally from its trial point estimate and 95% CI. rPFS and OS parameter draws were sampled independently across endpoints. The CEAC was generated across a WTP range of $0 to $500,000 per QALY in $10,000 increments. The summary probabilistic ICER is reported as the ratio of mean incremental cost to mean incremental QALY across all iterations, not as the mean of individual iteration ICER ratios. OWSA ranges were chosen to span the plausible uncertainty for each parameter: cost parameters were varied by plus or minus 20% to 50% (wider for less certain inputs such as generic drug cost, narrower for CMS-scheduled costs), utilities across their published confidence limits, the OS hazard ratio across its trial 95% confidence interval (0.71 to 1.15), and the time horizon and discount rate across conventional reference-case bounds (10 years to lifetime; 0% to 5%). A coefficient of variation of 0.10 was applied to cost parameters as a conventional, moderately informative choice reflecting that the central cost inputs are known with relatively high precision; because the incremental cost exceeds the maximum cost-effective cost by more than an order of magnitude, a wider cost distribution does not change the probabilistic result.

## Results

### Model inputs

Model input parameters are summarized in Table 2. The placebo OS arm was best fit by generalized gamma among plausible-tail distributions after excluding log-normal and log-logistic for implausible 40-year tails; the AIC advantage over Weibull was small (1465.70 vs 1466.75) and BIC differences were negligible, so this residual structural uncertainty was explored directly in the OS extrapolation scenarios (Table 1). AIC and BIC values for all six OS distributions in both arms, and the selected rPFS distributions per arm, are presented in Table 2.

**Table 2:** Model Input Parameters (Base Case).

a. Survival model selection (AIC / BIC)						
**Dataset**	**Distribution**	**AIC**	**BIC**	**S(40yr)**	**Plausible?**	**Selected?**
rPFS capivasertib	Exponential	1769.62	1773.85	0.0000	Y	N
rPFS capivasertib	Weibull	1767.59	1776.04	0.0000	Y	N
rPFS capivasertib	Log-normal	1772.66	1781.12	0.0304	N	N
rPFS capivasertib	Log-logistic	1762.28	1770.73	0.0243	N	Y
rPFS capivasertib	Gompertz	1771.39	1779.85	0.0000	Y	N
rPFS capivasertib	Generalized gamma	1767.05	1779.74	0.0005	Y	N
rPFS placebo	Exponential	1992.30	1996.52	0.0000	Y	N
rPFS placebo	Weibull	1984.57	1993.02	0.0000	Y	N
rPFS placebo	Log-normal	1960.36	1968.81	0.0061	Y	Y
rPFS placebo	Log-logistic	1967.86	1976.31	0.0118	N	N
rPFS placebo	Gompertz	1994.25	2002.70	0.0000	Y	N
rPFS placebo	Generalized gamma	1960.90	1973.57	0.0205	N	N
OS capivasertib	Exponential	1404.71	1408.94	0.0034	Y	N
OS capivasertib	Weibull	1404.42	1412.88	0.0002	Y	N
OS capivasertib	Log-normal	1417.15	1425.61	0.1328	N	N
OS capivasertib	Log-logistic	1405.83	1414.29	0.0702	N	N
OS capivasertib	Gompertz	1404.20	1412.66	0.0000	Y	N
OS capivasertib	Generalized gamma	1406.06	1418.75	0.0000	Y	N
OS placebo	Exponential	1476.99	1481.22	0.0020	Y	N
OS placebo	Weibull	1466.75	1475.20	0.0000	Y	N
OS placebo	Log-normal	1467.39	1475.84	0.0463	N	N
OS placebo	Log-logistic	1462.69	1471.14	0.0304	N	N
OS placebo	Gompertz	1475.32	1483.77	0.0000	Y	N
OS placebo	Generalized gamma	1465.70	1478.37	0.0035	Y	Y
**b. Survival model parameters (selected distributions; time in months)**						
**Arm / endpoint**		**Distribution**	**Fitted parameters (estimate, SE)**			**Source**
rPFS capivasertib		Log-logistic	shape 1.3398 (SE 0.0627); scale 30.4531 mo (SE 0.0788)			Fizazi et al. [5], Fig 3A (flexsurv)
rPFS placebo		Log-normal	meanlog 3.2246 (SE 0.0706); sdlog 1.1764 (SE 0.0522)			Fizazi et al. [5], Fig 3A (flexsurv)
OS placebo		Generalized gamma	mu 4.0285 (SE 0.1000); sigma 1.0174 (SE 0.1570); Q 0.4955 (SE 0.2564)			CAPItello-281 Fig 3A (flexsurv)
OS capivasertib		Proportional hazards (derived)	S_OS_capi(t) = S_OS_placebo(t) ^ 0.90; OS HR 0.90 (95% CI 0.71 to 1.15)			CAPItello-281 OS HR; PH base case
**c. Costs (2025 USD, per 4-week cycle unless noted)**						
**Parameter**		**Base-case value**	**OWSA range**		**PSA distribution**	**Source**
Capivasertib (capi arm, PFS)		$23,762.00	$16,633.40 to $30,890.60		Gamma (CV 0.10)	Publicly available list-price proxy, Drugs.com price guide
Generic abiraterone (both, PFS)		$92.91 ($101.00/mo)	$46.46 to $139.37		Gamma (CV 0.10)	CMS NADAC Apr 2026
Prednisone 5 mg (both, PFS)		$13.80 ($15.00/mo)	Not varied		Fixed	Generic list price
ADT leuprolide (both, PFS)		$162.32 ($176.45/mo)	$129.86 to $194.78		Gamma (CV 0.10)	Medicare ASP J9217
Companion diagnostic (capi, one-time)		$107.55	Not varied		Fixed	CMS Physician Fee Schedule, CPT 88360, 2026
Glucose/HbA1c monitoring (capi, PFS)		$6.59/cycle + $11.79 once	Not varied		Fixed	TRUQAP PI 06/2026; CPT 82947 $3.93 monthly + 83036 $9.71 quarterly; 2026 CLFS eff 2026-01-01
PPS / mCRPC care (both)		$9,652.11 ($10,492.36/mo)	$7,721.69 to $11,582.53		Gamma (CV 0.10)	Kaye 2024, J Manag Care Spec Pharm 30(7):684-697 (PMID 38950154); all-cause mCRPC $9,717 PPPM (2022 USD) inflated to 2025 USD by BEA PCE health care price index (factor 1.0798)
AE mgmt: hyperglycemia (unit)		$2,250.00	$1,800.00 to $2,700.00		Gamma (CV 0.20)	Assumption (grade >=3 event mgmt); varied widely in OWSA/PSA
AE mgmt: diarrhea (unit)		$1,000.00	$800.00 to $1,200.00		Gamma (CV 0.20)	Assumption (grade >=3 event mgmt); varied widely in OWSA/PSA
AE mgmt: cutaneous (unit)		$550.00	$440.00 to $660.00		Gamma (CV 0.20)	Assumption (grade >=3 event mgmt); varied widely in OWSA/PSA
**d. Adverse event rates (grade >=3; safety set n=503 per arm)**						
**AE**	**Capi rate**	**Placebo rate**	**Incremental**		**PSA distribution**	**Source**
Hyperglycemia	0.103 (52/503)	0.006 (3/503)	0.097		Beta(52,451); Beta(3,500)	Fizazi 2026 safety
Diarrhea	0.062 (31/503)	0.004 (2/503)	0.058		Beta(31,472); Beta(2,501)	Fizazi 2026 safety
Cutaneous	0.123 (62/503)	0.002 (1/503)	0.121		Beta(62,441); Beta(1,502)	Fizazi 2026 safety
**e. Health-state utilities and disutilities**						
**Parameter**		**Base-case value**	**OWSA range**		**PSA distribution**	**Source**
PFS utility (both arms)		0.80	0.72 to 0.85		Beta (CV 0.10)	Agarwal 2019 (TITAN, EQ-5D-5L) [23]
PPS (mCRPC) utility (both arms)		0.63	0.58 to 0.75		Beta (CV 0.10)	Agarwal 2019 (TITAN, EQ-5D-5L), progression state [23]
AE disutility: hyperglycemia		0.075 (1 cycle)	0.05 to 0.10		Fixed	Lloyd 2006 [24]; Nafees 2008 [25]
AE disutility: diarrhea		0.065 (1 cycle)	0.05 to 0.08		Fixed	Lloyd 2006 [24]; Nafees 2008 [25]
AE disutility: cutaneous		0.045 (1 cycle)	0.03 to 0.06		Fixed	Lloyd 2006 [24]; Nafees 2008 [25]
PRO decrement (capi PFS; exploratory, S2 only)		-0.04	0 to -0.07		Not in base case	FACT-P HR 1.43 (ASCO GU 2026)
**f. Model settings**						
**Setting**			**Value**			
Time horizon			40 years (lifetime)			
Cycle length			4 weeks (28 days)			
Discount rate			3% per year (costs and QALYs)			
Half-cycle correction			Yes (trapezoidal)			
Perspective			US third-party payer			
Background mortality			CDC 2022 US male life table floor; start age 68			

AIC, Akaike information criterion; BIC, Bayesian information criterion; AE, adverse event; CV, coefficient of variation; OWSA, one-way sensitivity analysis; PSA, probabilistic sensitivity analysis; PFS, progression-free survival; PPS, post-progression survival. Data repository: https://doi.org/10.17605/OSF.IO/EWXQT.

### Base case

In the base case (Table 3), the capivasertib arm accrued 4.103 discounted QALYs at a total discounted cost of $1,455,507, compared with 3.703 discounted QALYs at $207,230 for the abiraterone comparator. The incremental cost was $1,248,277, driven primarily by capivasertib acquisition costs during PFS. Incremental QALYs were 0.400, yielding an ICER of $3,120,720 per QALY. Net monetary benefit at a WTP threshold of $150,000 per QALY was approximately negative $1,188,000.

**Table 3:** Base-Case Cost-Effectiveness Results.

Outcome	Capivasertib	Placebo	Incremental
Total cost (disc, 2025 USD)	1,455,507	207,230	1,248,277
Total QALYs (disc)	4.103	3.703	0.400
Mean OS, yrs (undisc)	6.39	5.79	0.6
Mean PFS, yrs (undisc)	4.89	3.93	0.96
ICER ($/QALY)			3,120,720
NMB @ $100k			-1,208,277
NMB @ $150k			-1,188,277
**b. Mean Discounted Costs per Arm by Category (2025 USD)**			
**Cost category**	**Capivasertib arm**	**Comparator arm**	**Incremental**
Capivasertib drug	$1,280,173	$0	$1,280,173
Abiraterone	$5,006	$4,130	$875
Prednisone	$743	$613	$130
ADT (leuprolide)	$8,745	$7,216	$1,529
Companion diagnostic	$108	$0	$108
Glucose/HbA1c monitoring	$367	$0	$367
AE management	$343	$0	$343
PPS / post-progression care	$160,022	$195,271	-$35,249
**TOTAL**	**$1,455,507**	**$207,230**	**$1,248,277**

Costs discounted at 3%/year over 40 years. Monitoring applies to the capivasertib arm only. Incremental total reconciles to the engine output to the dollar. Data repository: https://doi.org/10.17605/OSF.IO/EWXQT.

### One-way sensitivity analysis

The tornado diagram (Fig 3) plots incremental NMB at $150,000 per QALY and shows that capivasertib list price was by far the most influential parameter, followed by the discount rate, time horizon, and the OS hazard ratio. Incremental NMB remained substantially negative (below approximately negative $800,000) across the full plausible range of every parameter, including the most favorable value of capivasertib price tested (negative 30%). No single parameter, varied across its full range, produced positive NMB at any threshold up to $500,000 per QALY.

**Fig 3:**
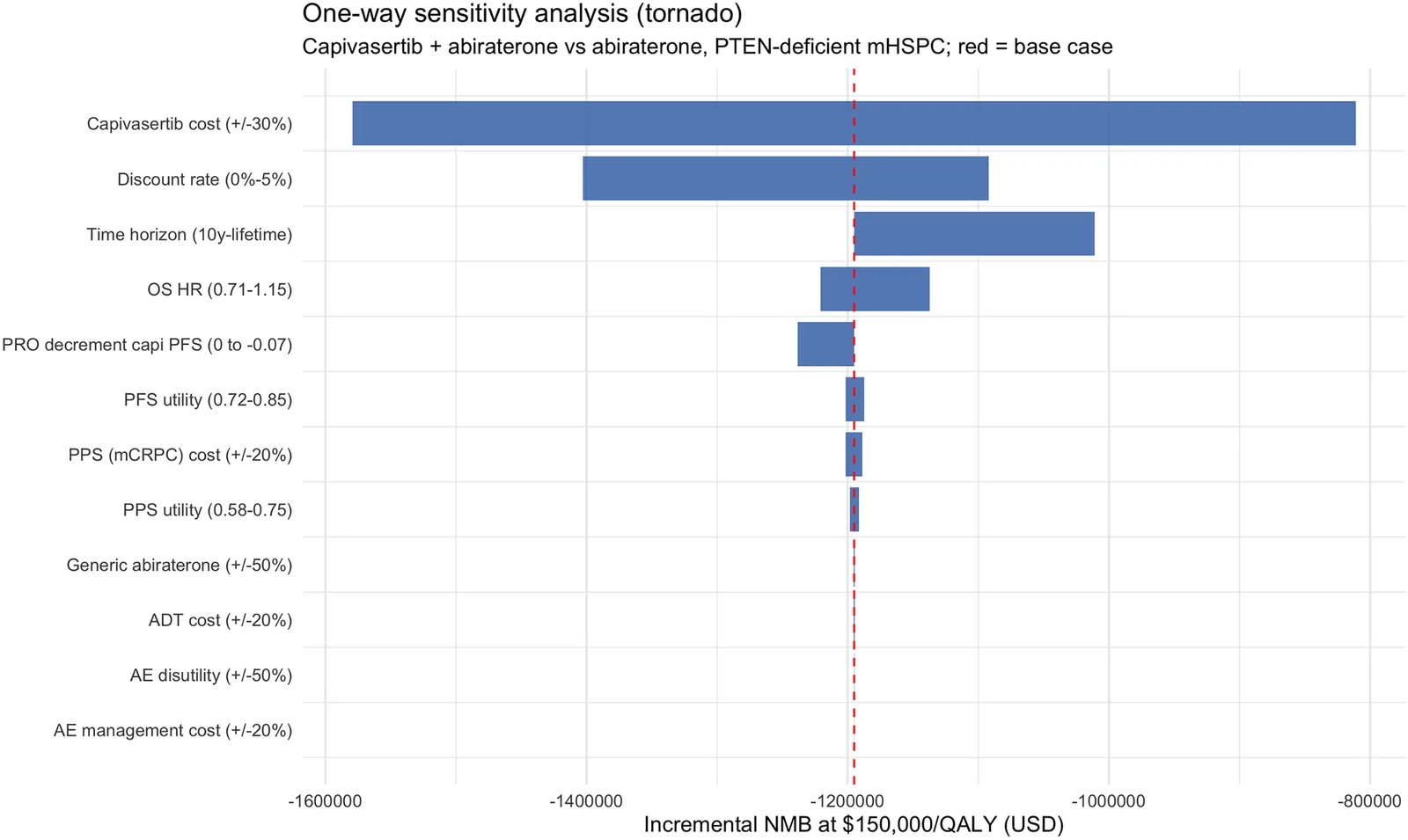
One-way sensitivity analysis tornado diagram. Bars show incremental net monetary benefit (NMB) at a willingness-to-pay of $150,000 per QALY as each parameter is varied across its plausible range (parameter ranges in parentheses); the dashed line marks the base-case NMB. NMB is plotted rather than the ICER because incremental QALYs cross zero under some parameter values, making ICERs unstable. Incremental NMB remained negative across the full range of every parameter.

### Overall survival extrapolation scenarios

Across the eight pre-specified OS extrapolation scenarios, capivasertib was either dominated or produced an ICER exceeding $1.35 million per QALY (Table 1). The most favorable scenario was log-normal independent arms (ICER $1,359,445 per QALY). Two scenarios produced negative incremental QALYs, in which capivasertib was dominated: the generalized gamma independent-arms fit and the per-arm best-AIC artifact scenario (Gompertz capivasertib, log-logistic placebo), the latter reflecting the distribution-mixing tail artifact documented in the preregistration and reported here for transparency. The PH base-case ICER of $3,120,720 per QALY was intermediate among the scenarios yielding positive incremental QALYs, consistent with its preregistered role as the methodological central estimate rather than a result-optimizing choice.

### Probabilistic sensitivity analysis

In PSA (n = 10,000; Fig 4), the ratio of mean incremental cost to mean incremental QALY was $3,148,744 per QALY. The probability of cost-effectiveness was 0% at WTP thresholds of $100,000, $150,000, $200,000, and $500,000 per QALY (Fig 5). Of the 10,000 iterations, 8,646 (86.46%) fell in the northeast quadrant of the cost-effectiveness plane (higher cost, positive incremental QALY) and 1,354 (13.54%) in the northwest quadrant (higher cost, negative incremental QALY). Because a subset of iterations yielded a negative incremental QALY, the distribution of individual iteration ICER ratios is uninformative, and the cost-effectiveness acceptability curve and plane provide the appropriate characterization of decision uncertainty.

**Fig 4:**
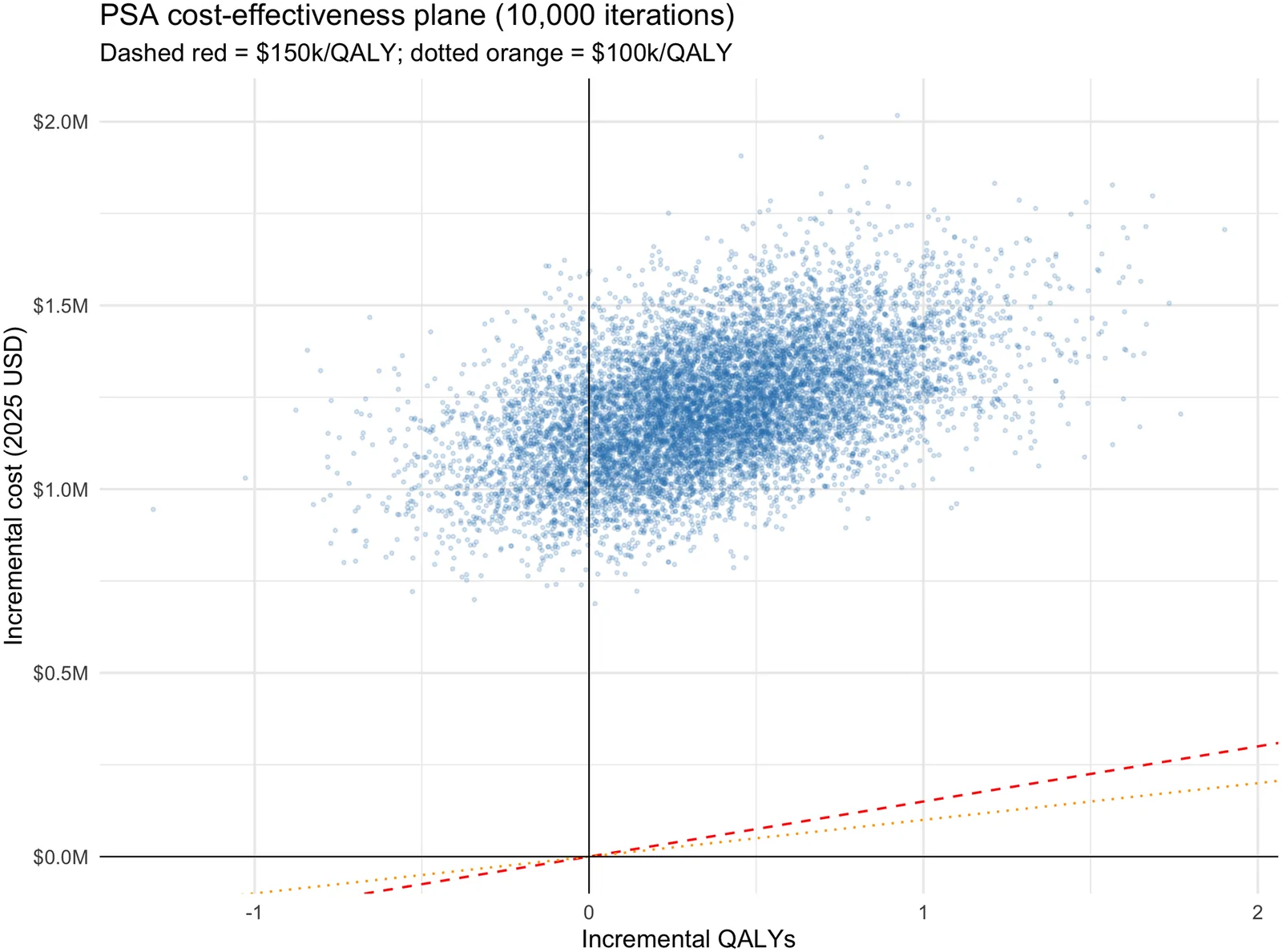
Probabilistic sensitivity analysis cost-effectiveness plane (n = 10,000 iterations). Incremental cost (y-axis) versus incremental QALYs (x-axis); willingness-to-pay reference lines at $100,000 (dotted orange) and $150,000 (dashed red) per QALY. All iterations lie above the WTP lines (higher cost without cost-effective benefit); approximately 13.54% fall left of the vertical axis, reflecting iterations with negative incremental QALYs under immature-OS sampling.

**Fig 5:**
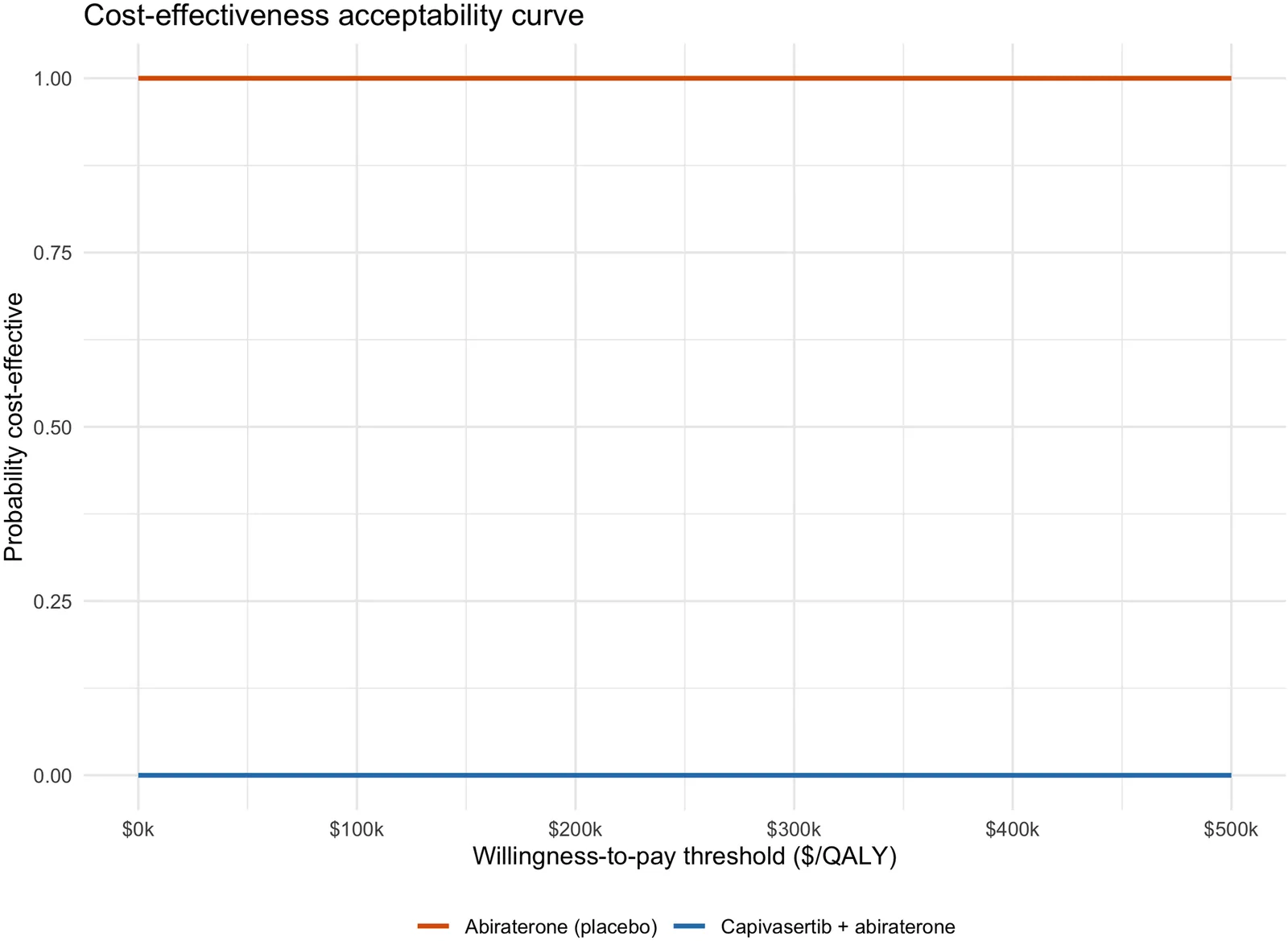
Cost-effectiveness acceptability curve (CEAC) showing the probability that each strategy is cost-effective across willingness-to-pay thresholds from $0 to $500,000 per QALY (n = 10,000 PSA iterations). Capivasertib had a 0% probability of being cost-effective across the entire range, so its curve lies on the x-axis and the comparator curve lies at 1.0; this reflects that no PSA iteration favored capivasertib at any threshold up to $500,000 per QALY, not a plotting error.

### Scenario analyses

Full scenario analysis results are presented in Table 4. Scenario S1 (brand abiraterone list price instead of generic) produced a minimally different ICER ($3,249,560 per QALY), reflecting the symmetry of abiraterone costs across both arms. Scenario S2 (exploratory PRO-informed PFS utility decrement of −0.04 in the capivasertib arm) worsened the ICER to $5,316,451 per QALY by reducing the incremental QALY gain. Scenario S3 (10-year time horizon) yielded an ICER of $4,426,676 per QALY. Under scenario S4 (WTP threshold of $200,000 per QALY), net monetary benefit remained negative (approximately negative $1.17 million). No scenario brought capivasertib near cost-effectiveness.

**Table 4:** Pre-Specified Scenario Analyses. ICERs are calculated from unrounded incremental cost and QALY values; recomputation from the rounded values displayed may differ by up to a few thousand dollars per QALY.

Scenario	Incremental cost	Incremental QALY	ICER ($/QALY)	NMB at $150k
Base case (PH HR = 0.90)	$1,248,277	0.400	$3,120,720	-$1,188,277
S1: Brand abiraterone (Zytiga list price).	$1,299,813	0.400	$3,249,560	-$1,239,813
S2: PRO-adjusted PFS utility (capi −0.04).	$1,248,277	0.235	$5,316,451	-$1,213,058
S3: 10-year time horizon.	$1,042,132	0.235	$4,426,676	-$1,006,819
S4: WTP $200k/QALY (NMB at 200k).	$1,248,277	0.400	$3,120,720	-$1,168,278

NMB, net monetary benefit (at $150,000/QALY except S4 at $200,000/QALY). Data repository: https://doi.org/10.17605/OSF.IO/EWXQT.

## Discussion

This preregistered PSM found that capivasertib plus abiraterone and prednisone is not cost-effective for PTEN-deficient mHSPC at current US list pricing from a third-party payer perspective. The base-case ICER of $3,120,720 per QALY substantially exceeds conventional US willingness-to-pay thresholds. The result was robust: across the prespecified OS extrapolation scenarios, capivasertib was either dominated or produced ICERs exceeding $1,350,000 per QALY, and the PSA probability of cost-effectiveness was 0% across a $0 to $500,000 per QALY range. These findings are consistent with CADTH and CDA-AMC assessments of capivasertib in breast cancer, which also concluded the drug did not represent good value at list price [27].

The result is structurally driven by two features: a high incremental drug acquisition cost (approximately $309,000 per patient-year at list price during the PFS state) and a small incremental QALY gain (0.400 over a lifetime horizon). The modest rPFS HR of 0.81 and the nonsignificant, immature OS HR of 0.90 together constrain the QALY gain regardless of OS extrapolation assumptions. A formal threshold analysis illustrates the magnitude of the gap: at a WTP of $150,000 per QALY and the base-case incremental gain of 0.400 QALYs, the maximum cost-effective incremental lifetime cost is approximately $60,000, against a modeled incremental cost of $1,248,277. Achieving cost-effectiveness at $150,000 per QALY would therefore require an incremental-cost reduction of roughly 95%; even in a hypothetical scenario in which the incremental QALY gain doubled, the required reduction would still exceed 90%. These are structural, not marginal, barriers.

The OS modeling challenge in this analysis requires detailed explanation. At the time of FDA approval, OS was 26.4% mature (HR 0.90; 95% CI 0.71 to 1.15; p = 0.401) [5]. AIC values across six parametric families differed by less than 1 point, providing no statistical basis for single-family selection, while 40-year restricted mean survival estimates ranged implausibly from 55 to 151 months across families. Independent per-arm best-AIC fitting produced a clinically implausible inversion driven by a small-sample terminal step artifact rather than trial signal. The preregistered PH base case was selected prior to any ICER calculation and is documented in the OSF preregistration. It preserves the trial OS point estimate under a proportional hazards assumption while avoiding the unstable tail artifact, consistent with NICE DSU TSD 14 guidance for immature OS data [17,18]. This approach does not assert neutrality. Rather it still imposes proportional hazards and applies an immature, nonsignificant point estimate, while avoiding manufacturing a survival benefit through distribution selection.

The patient-reported outcomes findings from George et al. [26] add a further complicating dimension. Time to clinically meaningful decline in FACT-P physical well-being favored the placebo arm (HR 1.43), and the preregistered exploratory PRO scenario worsened the ICER, indicating that adverse-event-related quality-of-life effects in the capivasertib arm compound rather than attenuate the cost-effectiveness challenge.

The use of list prices rather than negotiated net prices merits discussion. Confidential rebates, increasingly common for oncology agents under Inflation Reduction Act dynamics, may reduce actual payer cost below list price. However, the price reduction required to achieve cost-effectiveness appears sufficiently large (exceeding 90% under optimistic OS assumptions) that a rebate alone is unlikely to close the gap. Budget impact and outcomes-based contracting tied to mature OS data represent plausible payer strategies given this profile.

Limitations of this analysis include the following. First, OS was immature at analysis (26.4% maturity), and the OS HR was nonsignificant; all survival extrapolations carry substantial uncertainty that will only be resolved with mature data (projected study completion March 2027). Second, health state utilities were derived from the TITAN EQ-5D-5L analysis [23] rather than CAPItello-281-specific EQ-5D data, which were not available at time of analysis. Third, the model assumes full dose intensity and does not capture dose reductions or treatment interruptions that may reduce drug costs in practice. Fourth, the model assumes perfect PTEN IHC diagnostic performance [28]; real-world intratumoral heterogeneity may reduce the proportion of patients appropriately classified as PTEN-deficient. Fifth, post-progression therapy patterns are simplified and may not reflect rapidly evolving mHSPC-to-mCRPC sequencing. Sixth, this analysis takes a US third-party payer perspective; patient copay burden at list prices of approximately $24,000 per 28-day cycle represents a substantial access barrier not captured in the ICER. Seventh, capivasertib’s list price was held constant over the time horizon. This is conservative against capivasertib, because loss of exclusivity and subsequent generic or biosimilar entry would reduce its acquisition cost in later years; however, because treatment is confined to the progression-free phase (median rPFS approximately 33 months) and costs are concentrated early and discounted at 3% per year, this assumption has limited effect on incremental cost and does not alter the conclusion.

## Conclusions

Capivasertib plus abiraterone and prednisone is not cost-effective for PTEN-deficient mHSPC at current US list pricing. The conclusion that capivasertib was not cost-effective was robust across the eight prespecified OS extrapolation scenarios and across 10,000 PSA iterations. Mature OS data from CAPItello-281 and a major reduction in capivasertib acquisition cost would likely be required for cost-effectiveness at conventional US willingness-to-pay thresholds.

## Supporting information

S1 Table Auditable model input parameters (survival model selection and fitted parameters, costs, adverse event rates, utilities and disutilities, and model settings). Includes AIC/BIC for all six parametric distributions across all four reconstructed datasets.
(DOCX)

S2 Table Mean discounted costs per arm by category (2025 USD). Cost disaggregation reconciling to the base-case incremental cost.
(DOCX)
