# Supplementary material for: Cost-effectiveness of capivasertib plus abiraterone for PTEN-deficient metastatic hormone-sensitive prostate cancer in the United States
Source: PLoS One. 2026 Oct 26;21(10):e0353373. doi: 10.1371/journal.pone.0353373 (PMC13704799; doi:10.1371/journal.pone.0353373)
Supplement: Supplementary file 1 [file pone.0353373.s001.docx]

**S1 Table.** Auditable model input parameters. Survival model selection (AIC/BIC for six parametric distributions across four reconstructed datasets), fitted survival parameters, costs, adverse event rates, health-state utilities and disutilities, and model settings. Cost-effectiveness analysis of capivasertib plus abiraterone and prednisone versus abiraterone and prednisone (with ADT) for PTEN-deficient mHSPC; anchor trial CAPItello-281; three-state partitioned survival model; US third-party payer perspective; 2025 USD.

**S1a. Survival model selection (AIC / BIC)**

| **Dataset** | **Distribution** | **AIC** | **BIC** | **S(40yr)** | **Plausible? (Y/N)** | **Selected? (Y/N)** |
| --- | --- | --- | --- | --- | --- | --- |
| rPFS capivasertib | Exponential | 1769.62 | 1773.85 | 0.0000 | Y | N |
| rPFS capivasertib | Weibull | 1767.59 | 1776.04 | 0.0000 | Y | N |
| rPFS capivasertib | Log-normal | 1772.66 | 1781.12 | 0.0304 | N | N |
| rPFS capivasertib | Log-logistic | 1762.28 | 1770.73 | 0.0243 | N | Y |
| rPFS capivasertib | Gompertz | 1771.39 | 1779.85 | 0.0000 | Y | N |
| rPFS capivasertib | Generalized gamma | 1767.05 | 1779.74 | 0.0005 | Y | N |
| rPFS placebo | Exponential | 1992.30 | 1996.52 | 0.0000 | Y | N |
| rPFS placebo | Weibull | 1984.57 | 1993.02 | 0.0000 | Y | N |
| rPFS placebo | Log-normal | 1960.36 | 1968.81 | 0.0061 | Y | Y |
| rPFS placebo | Log-logistic | 1967.86 | 1976.31 | 0.0118 | N | N |
| rPFS placebo | Gompertz | 1994.25 | 2002.70 | 0.0000 | Y | N |
| rPFS placebo | Generalized gamma | 1960.90 | 1973.57 | 0.0205 | N | N |
| OS capivasertib | Exponential | 1404.71 | 1408.94 | 0.0034 | Y | N |
| OS capivasertib | Weibull | 1404.42 | 1412.88 | 0.0002 | Y | N |
| OS capivasertib | Log-normal | 1417.15 | 1425.61 | 0.1328 | N | N |
| OS capivasertib | Log-logistic | 1405.83 | 1414.29 | 0.0702 | N | N |
| OS capivasertib | Gompertz | 1404.20 | 1412.66 | 0.0000 | Y | N |
| OS capivasertib | Generalized gamma | 1406.06 | 1418.75 | 0.0000 | Y | N |
| OS placebo | Exponential | 1476.99 | 1481.22 | 0.0020 | Y | N |
| OS placebo | Weibull | 1466.75 | 1475.20 | 0.0000 | Y | N |
| OS placebo | Log-normal | 1467.39 | 1475.84 | 0.0463 | N | N |
| OS placebo | Log-logistic | 1462.69 | 1471.14 | 0.0304 | N | N |
| OS placebo | Gompertz | 1475.32 | 1483.77 | 0.0000 | Y | N |
| OS placebo | Generalized gamma | 1465.70 | 1478.37 | 0.0035 | Y | Y |

*Plausible-tail rule: S(40yr) < 0.01 (a patient surviving to age >=108 is implausible). rPFS capivasertib Log-logistic is selected per the prespecified per-arm best-AIC rule despite a flagged tail (S40=0.0243); in the PSM, S(rPFS) is capped at S(OS), so the tail is bounded structurally. OS capivasertib in the base case is NOT a standalone fit: it is derived by proportional hazards from the placebo OS fit (see 1b), so no OS capivasertib distribution is marked Selected. Abbreviations: AIC, Akaike information criterion; BIC, Bayesian information criterion; ADT, androgen deprivation therapy; AE, adverse event; CV, coefficient of variation; ICER, incremental cost-effectiveness ratio; OWSA, one-way sensitivity analysis; PFS, progression-free survival; PPS, post-progression survival; PSA, probabilistic sensitivity analysis; QALY, quality-adjusted life-year; WTP, willingness-to-pay.*

**S1b. Survival model parameters (selected base-case distributions; time in months)**

| **Arm / endpoint** | **Distribution** | **Fitted parameters (estimate, standard error)** | **Source** |
| --- | --- | --- | --- |
| rPFS capivasertib | Log-logistic | shape = 1.3398 (SE 0.0627, log scale); scale = 30.4531 mo (SE 0.0788, log scale) | Reconstructed IPD, CAPItello-281 Fig 2A (flexsurv) |
| rPFS placebo | Log-normal | meanlog = 3.2246 (SE 0.0706); sdlog = 1.1764 (SE 0.0522, log scale) | Reconstructed IPD, CAPItello-281 Fig 2A (flexsurv) |
| OS placebo | Generalized gamma | mu = 4.0285 (SE 0.1000); sigma = 1.0174 (SE 0.1570, log scale); Q = 0.4955 (SE 0.2564) | Reconstructed IPD, CAPItello-281 Fig 3A (flexsurv) |
| OS capivasertib | Proportional hazards (derived) | S_OS_capi(t) = S_OS_placebo(t) ^ 0.90; OS HR = 0.90 (95% CI 0.71-1.15), sampled lognormal in PSA | CAPItello-281 trial OS HR (Fizazi 2026); PH base case (user decision) |

*Standard errors are reported on the flexsurv estimation (transformed) scale, on which PSA draws are taken via multivariate normal sampling using the full variance-covariance matrix. Per-cycle OS hazard is floored at the CDC 2022 US male life-table hazard (cohort start age 68). Abbreviations: AIC, Akaike information criterion; BIC, Bayesian information criterion; ADT, androgen deprivation therapy; AE, adverse event; CV, coefficient of variation; ICER, incremental cost-effectiveness ratio; OWSA, one-way sensitivity analysis; PFS, progression-free survival; PPS, post-progression survival; PSA, probabilistic sensitivity analysis; QALY, quality-adjusted life-year; WTP, willingness-to-pay.*

**S1c. Costs (2025 USD, per 4-week cycle unless noted)**

| **Parameter** | **Base-Case Value** | **Range (for OWSA)** | **PSA Distribution** | **Source** |
| --- | --- | --- | --- | --- |
| Capivasertib (capivasertib arm, PFS) | $23,762.00 | $16,633.40 to $30,890.60 (+/-30%) | Gamma (CV 0.10) | Publicly available list-price proxy, Drugs.com price guide |
| Generic abiraterone (both arms, PFS) | $92.91 ($101.00/mo) | $46.46 to $139.37 (+/-50%) | Gamma (CV 0.10) | CMS NADAC, Apr 2026 (~$101/mo); applied to both arms |
| Prednisone 5 mg (both arms, PFS) | $13.80 ($15.00/mo) | Not varied (fixed) | Fixed (not sampled) | ~$15/mo (generic) |
| ADT leuprolide (both arms, PFS) | $162.32 ($176.45/mo) | $129.86 to $194.78 (+/-20%) | Gamma (CV 0.10) | Medicare ASP J9217 $176.45/mo (7.5 mg, 1-mo depot) |
| Companion diagnostic (capivasertib arm, one-time) | $107.55 | Not varied (fixed) | Fixed (not sampled) | CMS Physician Fee Schedule, CPT 88360, 2026 |
| Glucose/HbA1c monitoring (capivasertib arm, PFS) | $6.59/cycle + $11.79 one-time | Not varied (fixed) | Fixed (not sampled) | TRUQAP PI rev 06/2026 sec 5.1/2.2: fasting glucose CPT 82947 ($3.93) monthly + HbA1c CPT 83036 ($9.71) quarterly; 2026 CMS CLFS, effective 2026-01-01 |
| PPS / mCRPC care (both arms) | $9,652.11 ($10,492.36/mo) | $7,721.69 to $11,582.53 | Gamma (CV 0.10) | Kaye 2024, J Manag Care Spec Pharm 30(7):684-697 (PMID 38950154); all-cause mCRPC $9,717 PPPM (2022 USD) inflated to 2025 USD by BEA PCE health care price index (factor 1.0798) |
| AE management: grade >=3 hyperglycemia (unit) | $2,250.00 | $1,800.00 to $2,700.00 (+/-20%) | Gamma (CV 0.20) | Assumption, grade >=3 event management; varied widely in OWSA/PSA |
| AE management: grade >=3 diarrhea (unit) | $1,000.00 | $800.00 to $1,200.00 (+/-20%) | Gamma (CV 0.20) | Assumption, grade >=3 event management; varied widely in OWSA/PSA |
| AE management: grade >=3 cutaneous (unit) | $550.00 | $440.00 to $660.00 (+/-20%) | Gamma (CV 0.20) | Assumption, grade >=3 event management; varied widely in OWSA/PSA |

*Month-to-cycle conversion factor 28/30.4375 = 0.91992. NADAC, National Average Drug Acquisition Cost; ASP, average sales price; CPT, Current Procedural Terminology. AE management cost OWSA varied jointly (+/-20%). Abbreviations: AIC, Akaike information criterion; BIC, Bayesian information criterion; ADT, androgen deprivation therapy; AE, adverse event; CV, coefficient of variation; ICER, incremental cost-effectiveness ratio; OWSA, one-way sensitivity analysis; PFS, progression-free survival; PPS, post-progression survival; PSA, probabilistic sensitivity analysis; QALY, quality-adjusted life-year; WTP, willingness-to-pay.*

**S1d. Adverse event rates (grade >=3; safety analysis set n=503 per arm)**

| **AE** | **Capivasertib rate** | **Placebo rate** | **Incremental rate** | **PSA distribution** | **Source** |
| --- | --- | --- | --- | --- | --- |
| Hyperglycemia | 0.103 (52/503) | 0.006 (3/503) | 0.097 | Beta(52, 451) capi; Beta(3, 500) placebo | Fizazi 2026 safety table |
| Diarrhea | 0.062 (31/503) | 0.004 (2/503) | 0.058 | Beta(31, 472) capi; Beta(2, 501) placebo | Fizazi 2026 safety table |
| Cutaneous | 0.123 (62/503) | 0.002 (1/503) | 0.121 | Beta(62, 441) capi; Beta(1, 502) placebo | Fizazi 2026 safety table |

*Incremental rate = capivasertib minus placebo, floored at 0 in PSA. Beta parameters are derived from trial event counts (alpha = events, beta = n - events; n = 503 per arm). Abbreviations: AIC, Akaike information criterion; BIC, Bayesian information criterion; ADT, androgen deprivation therapy; AE, adverse event; CV, coefficient of variation; ICER, incremental cost-effectiveness ratio; OWSA, one-way sensitivity analysis; PFS, progression-free survival; PPS, post-progression survival; PSA, probabilistic sensitivity analysis; QALY, quality-adjusted life-year; WTP, willingness-to-pay.*

**S1e. Health-state utilities and disutilities**

| **Parameter** | **Base-Case Value** | **Range (for OWSA)** | **PSA Distribution** | **Source** |
| --- | --- | --- | --- | --- |
| PFS utility (both arms) | 0.80 | 0.72 to 0.85 | Beta (CV 0.10) | Agarwal 2019 (TITAN, EQ-5D-5L) [23] |
| PPS (mCRPC) utility (both arms) | 0.63 | 0.58 to 0.75 | Beta (CV 0.10) | Agarwal 2019 (TITAN, EQ-5D-5L), progression state [23] |
| AE disutility: hyperglycemia | 0.075 (1 cycle, 28 d) | 0.05 to 0.10 | Fixed in PSA | Lloyd 2006 [24]; Nafees 2008 [25] |
| AE disutility: diarrhea | 0.065 (1 cycle, 28 d) | 0.05 to 0.08 | Fixed in PSA | Lloyd 2006 [24]; Nafees 2008 [25] |
| AE disutility: cutaneous | 0.045 (1 cycle, 28 d) | 0.03 to 0.06 | Fixed in PSA | Lloyd 2006 [24]; Nafees 2008 [25] |
| PRO decrement (capivasertib PFS; exploratory, scenario S2 only) | -0.04 | 0 to -0.07 | Not in base case | FACT-P physical well-being HR 1.43 (ASCO GU 2026) |

*The PRO-informed PFS utility decrement of -0.04 is exploratory and applied to the capivasertib PFS utility in scenario S2 only; it is not part of the base case. AE disutilities are applied for one cycle (28 days). Abbreviations: AIC, Akaike information criterion; BIC, Bayesian information criterion; ADT, androgen deprivation therapy; AE, adverse event; CV, coefficient of variation; ICER, incremental cost-effectiveness ratio; OWSA, one-way sensitivity analysis; PFS, progression-free survival; PPS, post-progression survival; PSA, probabilistic sensitivity analysis; QALY, quality-adjusted life-year; WTP, willingness-to-pay.*

**S1f. Model settings**

| **Setting** | **Value** |
| --- | --- |
| Time horizon | 40 years (lifetime) |
| Cycle length | 4 weeks (28 days) |
| Discount rate | 3% per year (costs and QALYs) |
| Half-cycle correction | Yes (trapezoidal state membership) |
| Perspective | US third-party payer |
| Background mortality | CDC 2022 US male life table; per-cycle OS hazard floor; cohort start age 68 |

*Abbreviations: AIC, Akaike information criterion; BIC, Bayesian information criterion; ADT, androgen deprivation therapy; AE, adverse event; CV, coefficient of variation; ICER, incremental cost-effectiveness ratio; OWSA, one-way sensitivity analysis; PFS, progression-free survival; PPS, post-progression survival; PSA, probabilistic sensitivity analysis; QALY, quality-adjusted life-year; WTP, willingness-to-pay.*
