# Supplementary material for: Cost-effectiveness of capivasertib plus abiraterone for PTEN-deficient metastatic hormone-sensitive prostate cancer in the United States
Source: PLoS One. 2026 Oct 26;21(10):e0353373. doi: 10.1371/journal.pone.0353373 (PMC13704799; doi:10.1371/journal.pone.0353373)
Supplement: Supplementary file 2 [file pone.0353373.s002.docx]

**S2 Table.** Mean discounted costs per arm by category (2025 USD). Cost disaggregation reconciling to the base-case incremental cost of $1,251,611. Costs discounted at 3% per year over a 40-year horizon.

| **Cost category** | **Capivasertib arm ($)** | **Comparator arm ($)** | **Incremental ($)** |
| --- | --- | --- | --- |
| Capivasertib drug | $1,280,173 | $0 | $1,280,173 |
| Abiraterone | $5,006 | $4,130 | $875 |
| Prednisone | $743 | $613 | $130 |
| ADT (leuprolide) | $8,745 | $7,216 | $1,529 |
| Companion diagnostic | $108 | $0 | $108 |
| Glucose/HbA1c monitoring | $367 | $0 | $367 |
| AE management | $343 | $0 | $343 |
| PPS / post-progression care | $160,022 | $195,271 | -$35,249 |
| **TOTAL** | **$1,455,507** | **$207,230** | **$1,248,277** |

*Costs are discounted at 3% per year over a 40-year horizon. Drug, monitoring, and PPS costs are accrued on discounted state membership; companion diagnostic, the year-1 glucose front-load, and AE management are one-time costs applied in cycle 1. Glucose/HbA1c monitoring applies to the capivasertib arm only. Abbreviations: AIC, Akaike information criterion; BIC, Bayesian information criterion; ADT, androgen deprivation therapy; AE, adverse event; CV, coefficient of variation; ICER, incremental cost-effectiveness ratio; OWSA, one-way sensitivity analysis; PFS, progression-free survival; PPS, post-progression survival; PSA, probabilistic sensitivity analysis; QALY, quality-adjusted life-year; WTP, willingness-to-pay.*
